# How to Make the Ghosts in my Bedroom Disappear? Focused-Attention Meditation Combined with Muscle Relaxation (MR Therapy)—A Direct Treatment Intervention for Sleep Paralysis

**DOI:** 10.3389/fpsyg.2016.00028

**Published:** 2016-01-29

**Authors:** Baland Jalal

**Affiliations:** Behavioural and Clinical Neuroscience Institute, Department of Psychiatry, University of CambridgeCambridge, UK

**Keywords:** sleep paralysis, treatment intervention, focused inward-attention meditation, muscle relaxation, hypnogogic and hypnopompic hallucinations, attentional shifting, mindfulness

## Abstract

Sleep paralysis (SP) is a common state of involuntary immobility occurring at sleep onset or offset. It can include terrifying hypnogogic or hypnopompic hallucinations of menacing bedroom intruders. Unsurprisingly, the experience is associated with great fear and horror worldwide. To date, there exist no direct treatment intervention for SP. In this article, I propose for the first time a type of focused inward-attention meditation combined with muscle relaxation as a direct intervention to be applied during the attack, to ameliorate and possibly eliminate it (what could be called, meditation-relaxation or MR therapy for SP). The intervention includes four steps: (1) *reappraisal of the meaning of the attack*; (2) *psychological and emotional distancing*; (3) *inward focused-attention meditation;* (4) *muscle relaxation.* The intervention promotes attentional shift away from unpleasant external and internal stimuli (i.e., terrifying hallucinations and bodily paralysis sensations) unto an emotionally pleasant internal object (e.g., a positive memory). It may facilitate a relaxed meditative state characterized by a shift from sympathetic to parasympathetic dominance, associated with greater levels of alpha activity (which may lead to drowsiness and potentially sleep). The procedure may also reduce the initial panic and arousal that occur when realizing one is paralyzed. In addition, I present a novel Panic-Hallucination (PH) Model of Sleep Paralysis; describing how through escalating cycles of fear and panic-like autonomic arousal, a positive feedback loop is created that worsens the attack (e.g., leading to longer and more fearful episodes), drives content of hallucinations, and causes future episodes of SP. Case examples are presented to illustrate the feasibility of MR therapy for SP.

## Background

Sleep paralysis refers to a state of involuntary immobility occurring at sleep onset or offset (Hobson, 1995). During SP there is an inhibition of voluntary muscles. The dorsolateral pons and ventromedial medulla suppress skeletal muscle tone during rapid eye movement (REM) sleep, through inhibition of spinal motor neurons; mediated by glycine and GABA in the spinal cord (Brooks and Peever, 2012). In spite of the atonia, the sensory system is clear, and ocular and respiratory movements intact. Thus during the attack, the individual experiences a transient period of semi-consciousness coupled with bodily paralysis (Jalal and Hinton, 2013).

Sleep paralysis is one of the symptoms of narcolepsy (Jalal and Ramachandran, 2014). Narcolepsy is a rare autoimmune sleep disorder occurring in less than one percent of the population (Ohayon et al., 2002). SP often occurs without narcolepsy. Such “isolated” sleep paralysis (ISP) episodes occur in approximately 20% of the general population (for a review see, Sharpless and Doghramji, 2015). The criteria for recurring isolated sleep paralysis (RISP) is undergoing four or more episodes per year (Bell et al., 1988; Jalal and Hinton, 2015).

The perceptual activity of REM may become activated during SP. REM mentation can generate vivid sensory experiences, hypnogogic (predormital) or hypnopompic (post dormital) hallucinations in all sensory modalities. SP hallucinations include experiencing levitation and autoscopy, having an out-of-body experience (OBE), and seeing, hearing, and sensing the presence of menacing intruders in one’s bedroom (Cheyne et al., 1999; Jalal and Hinton, 2013; Jalal and Ramachandran, 2014).

Sleep paralysis is often associated with great fear and terror, and sometimes impending death (Hufford, 2005; Jalal and Hinton, 2013). Unsurprisingly, the experience has been linked to symptoms of anxiety and trauma (e.g., Jalal and Hinton, 2015). Supernatural accounts of the phenomenon are common across cultures, ranging from demonic and ghost attacks to space alien abduction (Wing et al., 1994; Arikawa et al., 1999; Hinton et al., 2005a; McNally and Clancy, 2005; Jalal et al., 2014b, 2015).

## Treatment Interventions for Sleep Paralysis

Notwithstanding the great fear associated with the experience, there are few treatments available for SP. To date, there are no published clinical trials or outcome data for treating SP. My colleague Devon Hinton has conducted some of the most extensive work in treating SP carried out among traumatized Cambodians (e.g., Hinton et al., 2005a,b). He specifically targeted sleep disturbances and anxiety symptoms which are implicated in the onset of SP (e.g., Stores, 1998), and also used psychoeducation about the nature of the attack and modified catastrophic cognitions about SP (e.g., “I will be paralyzed for life”). Another approach to psychoeducation about SP specifically focuses on the hallucinations by telling sufferers that supernatural accounts of SP are common (Ohaeri et al., 1992; see too, Sharpless and Barber, 2011). To the best of my knowledge, there are no direct treatment interventions available for SP that are applied during the actual attack.

## Panic-Hallucination (PH) Model of Sleep Paralysis

At the onset of an SP attack, the individual will feel the effects of REM respiration such as hypoxia and hypercapnia, occlusion of airways and shallow rapid breathing (Douglas, 1994). This might result in chest pressure, and suffocation and choking sensations; especially so when the individual’s attempts to control breathing, by breathing deeply, fail (Hishikawa and Shimizu, 1995; Cheyne et al., 1999). Somatic symptoms, coupled with the awareness that one is paralyzed, can activate a host of psychological symptoms; including fear and worry that are worsened by catastrophic cognitions about the attack (e.g., “I am dying”). This in turn may generate an amygdaloid fight-flight reaction and panic-like arousal. As the individual attempts to move to overcome the paralysis, somatic symptoms and arousal are exacerbated. That is, the execution of motor programs in the absence of dampening proprioceptive feedback can lead to heightened sensations of bodily tightness and pressure, and even pain and spasms in limbs (Cheyne et al., 1999; Jalal and Ramachandran, 2014). Moreover, the desynchrony between motor-execution and feedback from limbs, may lead to distortions in body image, causing bodily hallucinations such as phantom limbs, floating sensations, and OBEs. Such body image disturbances might also cause hallucinations of shadowy-figures. For example, Ramachandran and I recently proposed that the shadowy-figure often seen during SP arises due to a disturbance in the multisensory processing of body and self at the temporoparietal junction (see Jalal and Ramachandran, 2014). The content and interpretation of hallucinations, are driven by multiple processes including hypervigilance for threat, the emotion of fear, and somatic sensations such as pressure on the chest and limbs, and REM-induced sexual arousal (i.e., hence common “rape scenarios”; see Jalal et al., 2014a); these are then imbedded in the experiencer’s socio-cultural framework (e.g., as “space alien abduction” or a “demonic attack”).

In brief, through escalating cycles of fear and panic-like autonomic arousal (“worry attacks”) a positive feedback loop is created that worsens the attack. This cycle may also cause future episodes of SP as the individual acquires conditioned fear of the experience; leading to more nighttime awakening (due to increased nocturnal arousal and hyper-alertness to symptoms of paralysis) which predisposes to SP.

## The Hypothesis: Focused-Attention Meditation Combined with Muscle Relaxation as a Direct Treatment Intervention for SP

In light of this proposed model of SP attacks, as we shall see, meditation might constitute an efficacious direct treatment intervention for SP. One aspect of meditative practice includes maintaining attention toward chosen meditative objects (Wallace, 2005) and attending to beneficial thoughts (Gethin, 2011). For example, attention may be focused inward on an attentional object, and whenever distracted by external stimuli or inner thoughts, attention is brought back to the inner-object of focus. This in turn allows the person to be released from uncontrollable thoughts, and enter into a calm mental state free from distractions.

I propose that a type of focused inward-attention meditation combined with muscle relaxation be applied directly during SP as an intervention to ameliorate and possibly eliminate the attack (one could call this treatment approach, meditation-relaxation or MR therapy for SP). (As SP entails seconds to minutes of conscious awareness and a sense of agency, the intervention can readily be applied during the episode.) I specifically propose the following steps to be implemented. Step I: *reappraisal of the meaning of the attack*: at the onset of the attack, the individual should reappraise the meaning of his SP episode by telling himself that the experience is common, benign, and temporary, and that the hallucinations are a typical byproduct of REM mentation (i.e., dreaming). Eyes should remain closed throughout the SP episode and the person should stay calm and avoid movement. (Ideally, the individual would have received prior psychoeducation about the nature of SP and associated hallucinations.) Step II: *psychological and emotional distancing*: next, the individual should tell himself that since the experience is common, benign, and temporary, there is no reason to be afraid or worried. That in fact, fear and worry (catastrophizing the event) will only make the episode worse and possibly prolong it, and are unnecessary emotions. Step III: *inward focused-attention meditation*: the individual should then focus his attention inward on an emotionally salient positive object (e.g., a memory of a loved one or event, a hymn/prayer, God). He should sustain his full attention on this inner-object and engage it emotionally (i.e., reflect on all its positive aspects). Bodily symptoms and external stimuli (i.e., hallucinations) should be ignored, and whenever distracted, attention should be brought back to the inner-object of focus. Step IV: *Muscle relaxation*: while engaging in focused inward-attention meditation, the individual should relax his muscles and avoid flexing them; and avoid controlling breathing and under no circumstances attempt to move. He should adopt a non-judgmental attitude of acceptance toward physical symptoms.

It is recommended that the method be practiced regularly, even in the absence of SP. For example, the individual may lay down in the supine position [i.e., sleeping in the supine position predisposes to having SP (see, Dahmen and Kasten, 2001)], and go through each step, simulating an actual attack. This would make it easier for the individual to apply the treatment once SP occurs, and not become too overwhelmed by subjective fear (e.g., due to amygdala activation), and the unpleasant features of REM atonia and respiration.

In general, long-term meditation training, might be useful as prior preparation, and possibly increase the effectiveness of the intervention. It might help the individual reach a relaxed meditative state more rapidly during SP, and enable him to sustain attention away from threatening stimuli for longer periods; e.g., by improving attentional skills (e.g., Wadlinger and Isaacowitz, 2011), and the ability to regulate emotions (e.g., Ortner et al., 2007; Robins et al., 2012). Ongoing meditation training might be particularly useful as RISP often occurs in clinical populations with anxiety disorders; including panic disorder (Bell et al., 1986; Friedman and Paradis, 2002), generalized anxiety disorder and social anxiety disorder (Simard and Nielsen, 2005; Otto et al., 2006), and post-traumatic stress disorder (Hinton et al., 2005b). High rates of SP may arise from the disturbed sleep characteristic of these clinical populations (for details see Jalal and Hinton, 2015). In this respect, research has shown that long-term meditation practitioners spend more time in slow wave sleep (SWS) (Mason et al., 1997), which is of key importance as SWS is associated with reduced autonomic arousal, thereby improving quality of sleep.

## Possible Benefits of the Proposed Treatment Intervention

**FIGURE 1:**
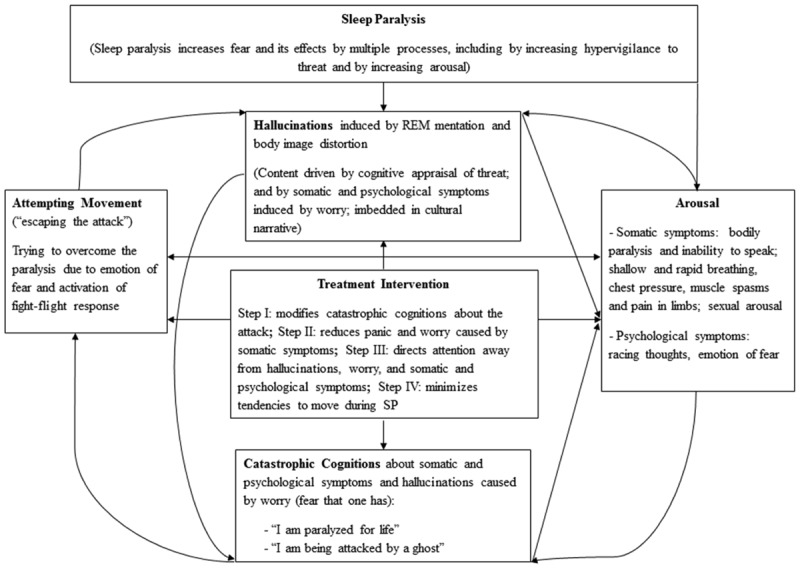
**The Panic-Hallucation (PH) Model of Sleep Paralysis; Moderated by the proposed treatement intervention**.

The proposed intervention may be advantageous for several reasons. By first reappraising the meaning of the attack and addressing catastrophic cognitions; and engaging in emotional distancing from the event, the initial panic and arousal that occur when realizing one is paralyzed may be reduced. Consistent with this, research has found that emotional regulation strategies such as reappraisal and cognitive distancing are associated with decreased amygdala activation (Beauregard et al., 2001; Lévesque et al., 2003). Moreover, as focused attention and emotion centeredness demand cognitive resources, attention directed away from threatening stimuli (e.g., hallucinations and paralysis sensations) may prevent worrying about the attack. Such focused meditation may in turn facilitate a relaxed meditative state and, possibly, a shift from sympathetic to parasympathetic dominance (e.g., Wu and Lo, 2008) (which might lead to drowsiness and potentially sleep). Inward-attention meditation in particular, has been found to be associated with lower levels of anxiety, feelings of calm and positive affect, and greater levels of alpha brain wave activity (Nagendra et al., 2012). Finally, muscle relaxation, whilst enhancing the relaxed state, may critically also, minimize tendencies to move during SP. This may reduce somatic symptoms such as chest pressure, muscle spasms, and prevent hallucinations that occur due to body image disturbances (e.g., OBEs and possibly human-like shadowy figures). Overall, the proposed treatment might minimize conditioned fear of the event and thus possibly resulting in fewer, shorter, and more benign future episodes of SP (see **Figure 1**).

## Case Examples: Practical Application of MR Therapy

### Case I. The Effect of MR Therapy on Hypnogogic and Hypnopompic Hallucinations

Joe, a man in his early thirties, experiences SP occasionally. His episodes can be terrifying, and may include vivid hallucinations. One afternoon when Joe was taking a nap, he suddenly found himself awake but unable to move. Petrified, he soon noticed that his body was floating in the air above his bed, and could hear voices of family members outside his bedroom. There in his room with him was also the “ghost” of his recently deceased friend. All the while experiencing these bizarre kinetic, auditory and visual hallucinations, he started to feel an overwhelming evil and threatening presence. The fear and horror would escalate with each passing moment. Joe realized that the many sensory inputs (i.e., hallucinations) were pulling his attention in multiple directions. So Joe resorted to focused inward-attention meditation: he closed his eyes and focused all his attention on a pleasant inner-object (e.g., a comforting thought), while completely ignoring all the hallucinations. Almost immediately thereafter, he noticed the terrifying hallucinations had vanished. His body had also become more relaxed. He soon awoke from his SP. [This curious case of meditation during SP is consistent with subjective reports that prayer can sometimes help thwart the attack (e.g., Jalal et al., 2014a). It is surprising that this individual’s hallucinations (unlike past SP episodes) vanished virtually abruptly when engaging in meditation during the attack.]

On a different occasion, Joe resorted to MR therapy to help thwart yet another SP attack. Joe had begun rehearsing the treatment, so it felt more natural to apply the steps. As the paralysis came over him, he felt an immense pressure on his chest, making breathing difficult; he closed his eyes, relaxed his muscles and stayed calm. He focused his attention, while repeating to himself that the incident was merely “caused by the brain, and therefore should not be feared” (i.e., his “attentional object”). This made him feel relaxed and confident and he was no longer frightened of “creatures” who might be lurking in his bedroom. So he did the “unthinkable”: he opened his eyes to face the menacing intruders! To his utter surprise, he saw the vague frame of three helpless and innocent-looking young boys in front of him. This was in stark contrast to his many past SP experiences, where he has encountered all kinds of horrors, including sexual molestation by demons. Joe believes that the combined meditation and relaxation—and the resulting positive emotions—has “transformed SP into something non-threatening” for him. He no longer fears SP! [It is consistent with the PH model of SP (see **Figure 1**), that MR therapy would eliminate or reduce SP hallucinations or modify their content from threatening (psychotic-like hallucination) into something more benign.]

### Case II. Severe RISP Associated with Anxiety, Worry, and PTSD Symptoms: Eight Weeks of MR Therapy

Jonah, who is in his late teens, experiences SP 3-5 times a week. His episodes are usually terrifying and include hallucinations of demonic possession. Unsurprisingly, his scores on self-report measures of trait anxiety [State-Trait Anxiety Inventory trait (STAI-T); Spielberger et al., 1983], pathological worry [The Penn State Worry Questionnaire (PSWQ); Meyer et al., 1990], and symptoms of PTSD [The PTSD Checklist (PCL); Weathers et al., 1993], suggest that he may possibly suffer from clinical levels of anxiety (but no formal diagnoses were made). Jonah applied MR therapy as often as possible, for about eight weeks to treat his SP, and also practiced the treatment steps outside SP, as preparation. It took a few SP episodes for him to get used to MR therapy, and overcome the panic and overwhelming fear that would ensue. While he felt some discomfort initially, by the end of the eight weeks he had become familiar with the treatment. As a result, his fear of SP disappeared; he noted when “you get used to the treatment, you know how to deal with it [SP] and approach SP in a more relaxed way” and, “when the fear is gone, you learn to appreciate it”; and generally, “you feel more grounded and less freaked out”. For Jonah, a key way of reappraising SP and distancing himself (psychologically and emotionally) from the event was to remind himself that, at this very moment there are millions of people who likewise experience SP. This made him feel connected to SP sufferers around the world. Jonah would focus on a “soothing” person he knew of, as part of his focused-attention meditation to help get his mind off worries. He stressed that he felt like a “white shield of energy” was protecting him from the “attacker” during SP, and this gave him a “sense of peace”. In brief, after approximately eight weeks of applying MR therapy as often as possible, Jonah’s conditioned fear of the experience disappeared, and the hallucinations weakened, and/or became more benign in nature. Also the number of episodes he would experience on a weekly basis dropped from around 3-5 to 2-3 episodes. Strikingly his symptoms of anxiety, pathological worry and PTSD dropped significantly from pre to post treatment: STAI-T from 58 to 49, the PSWQ from 58 to 40, and the PCL from 48 to 42; suggesting that his SP attacks might in part drive these symptoms.

## Concluding Remarks

Based on SP research experience in the United States, Europe and the Middle East (e.g., Jalal and Hinton, 2013, 2015, Jalal et al., 2014a,b, 2015), I have found that SP sufferers sometimes mention that relaxing during SP (e.g., relaxing muscles) helps thwart the attack. This is unsurprising given the mechanisms of the event presented above. Also SP sufferers report that engaging in prayer or religious recitation during the attack may eliminate the episode, and even cause the hallucinations of ominous “creatures” to vanish (e.g., Jalal et al., 2014a). While the causal role of supernatural forces cannot be disproven, another explanation (not necessarily excluding the former) is that prayers, hymns and religious recitations are analogues to meditation in several ways: they require attentional shifting away from threat unto a pleasant object, rely on emotion centeredness, focused attention and so on.

Only future experiments [e.g., using suitable controls and electroencephalographic (EEG)] can determine the feasibility of the hypothesis. Future research should also explore whether the intervention would yield different outcomes depending on whether the individual suffers from narcolepsy or anxiety disorders. Particularly in the latter group, as anxious individuals often have poor attentional control and emotion regulation skills (Eysenck et al., 2007; Amstadter, 2008; see also, Chambers et al., 2008), prior meditation training might be useful, or perhaps necessary, preparation before applying the treatment. Moreover, it remains to be examined to what extent cognitive mechanisms associated with meditation can influence the hallucinations seen during SP. For example, can they make the hallucinated shadowy-figure “disappear” (as it is reported that prayers may sometimes do)? One also wonders if the arrow may go in the opposite direction: whether the hallucinations of such shadowy-figures can be induced by attempting movement (causing disturbances in body image) and/or imagining their presence during the attack (activating REM mentation). This might not be inconceivable given that SP similar to lucid dreaming (i.e., being aware that one is dreaming and able to control aspects of one’s dream) may entail an activation of the dorsolateral prefrontal cortex (associated with willful action and a sense of agency, normally deactivated during REM sleep) as one begins to awaken. It is also consistent with the observation that some SP experiencers report that they can slide from SP into a lucid dreaming-like state and “astral project” (temporarily “leave” their body) during SP (e.g., Hufford, 1982). Being able to manipulate the content of one’s SP hallucinations may be therapeutic as it might convince the experiencer that the “shadowy-figure” arises due to events in the brain, can potentially be “self-created” and therefore is harmless (i.e., what one could call a type of “exposure therapy” for SP).

## Ethics Statement

The case examples were exempt from ethical approval [according to the NHS, Health Research Authority (HRA) guidelines, in the United Kingdom], as they were not part of a formal research study. The individuals gave consent and approved data to be published. Data were collected online and the cases applied the treatment in the convenience of their own home.

## Conflict of Interest Statement

The author declares that the research was conducted in the absence of any commercial or financial relationships that could be construed as a potential conflict of interest.
