# Erratum: How to Make the Ghosts in my Bedroom Disappear? Focused-Attention Meditation Combined with Muscle Relaxation (MR Therapy)—A Direct Treatment Intervention for Sleep Paralysis

**DOI:** 10.3389/fpsyg.2016.01194

**Published:** 2016-08-03

**Authors:** Frontiers Production Office

**Affiliations:** Frontiers Production Office, FrontiersSwitzerland

**Keywords:** sleep paralysis, treatment intervention, focused inward-attention meditation, muscle relaxation, hypnogogic and hypnopompic hallucinations, attentional shifting, mindfulness

Reason for Erratum:

Due to a typesetting error, the references of Sharpless and Barber (2011) and Sharpless and Doghramji (2015) were inadvertently interchanged.

In the section Background, second paragraph, the reference should be Sharpless and Barber (2011) and not Sharpless and Doghramji (2015) as published.

In the section Treatment Interventions For Sleep Paralysis, first paragraph, the reference should be Sharpless and Doghramji (2015) and not Sharpless and Barber (2011) as published.

The publisher apologizes for this mistake. This error does not change the scientific conclusions of the article in any way.
